# Genomic profiling of a patient with quadruple synchronous colorectal cancer: a case report

**DOI:** 10.1186/s12876-021-01935-x

**Published:** 2021-10-02

**Authors:** Xiongjie Jia, Xinyu Peng, Junjie Sun, Tao Zhang, Hengxue Lin, Tianliang Bai, Aimin Zhang

**Affiliations:** Department of Gastrointestinal Surgery, Affiliated Hospital of Hebei University, No. 212 Yuhua East Road, Baoding, 071000 Hebei China

**Keywords:** Synchronous colorectal cancer, Quadruple, Heterogeneity, Next-generation sequencing, Case report

## Abstract

**Background:**

Synchronous colorectal cancer (SCRC) is featured by the presence of multiple primary tumor lesions in a single patient at initial diagnosis. It is less common with the prevalence of approximately 3.5% among colorectal cancer (CRC). Some studies of SCRC have been performed in patients with two tumor lesions. However, SCRC cases with three or more tumor lesions were rare and remained to be investigated.

**Case presentation:**

In this case report, we presented a 56-year-old male SCRC case with quadruple tumor lesions which is rarely seen in clinical practice. After laparoscopic radical resection of sigmoid carcinoma and partial rectum resection, the four tumor samples were subjected to pathological evaluation and next-generation sequencing (NGS) based genetic profiling. The four tumor lesions included two adenocarcinomas with moderate differentiation at sigmoid colon and rectum respectively, a grade 1 neuroendocrine tumor (NET) at rectum and a high-grade intraepithelial neoplasia at ascending colon. Each tumor exhibited distinct histology types and mutation profiles. After surgical resection, the patient remained disease-free after four cycles of chemotherapy with oxaliplatin and capecitabine (XELOX).

**Conclusions:**

The tumor lesions in this case showed different pathological and genetic features which indicats the heterogeneity of SCRC. The genomic profilling might provide novel insights to understand SCRC at molecular level.

## Background

Synchronous colorectal cancer (SCRC) is a rare type of colorectal cancer (CRC) featured by simultaneous occurrence of multiple primary tumors in the same patient within six months since initial diagnosis. The prevalence of SCRC ranged from 1 to 8% among different populations [1]. A pooled data analysis of 39 studies estimated that the overall prevalence of SCRC was approximately 3.5% [1]. Patients with SCRC usually present two or three primary tumor lesions [2]. Some patients may have four or more tumors which are extremely rare in clinical practice [2]. Previous studies of SCRC were mainly performed in patients with two tumor lesions by comparing paired tumor samples to describe their clinical or molecular features [1–4]. SCRC cases with three or more tumors were rare and remained to be investigated [2, 5]. Herein, we reported a Chinese male patient with quadruple SCRC. Written informed consent was obtained from the patient. The patient received laparoscopic radical resection of sigmoid carcinoma and partial rectum resection. Capture-based targeted sequencing using a panel consisting of 168 cancer related genes was performed on each tumor to understand the heterogeneity of SCRC.

## Case presentation

**Fig. 1:**
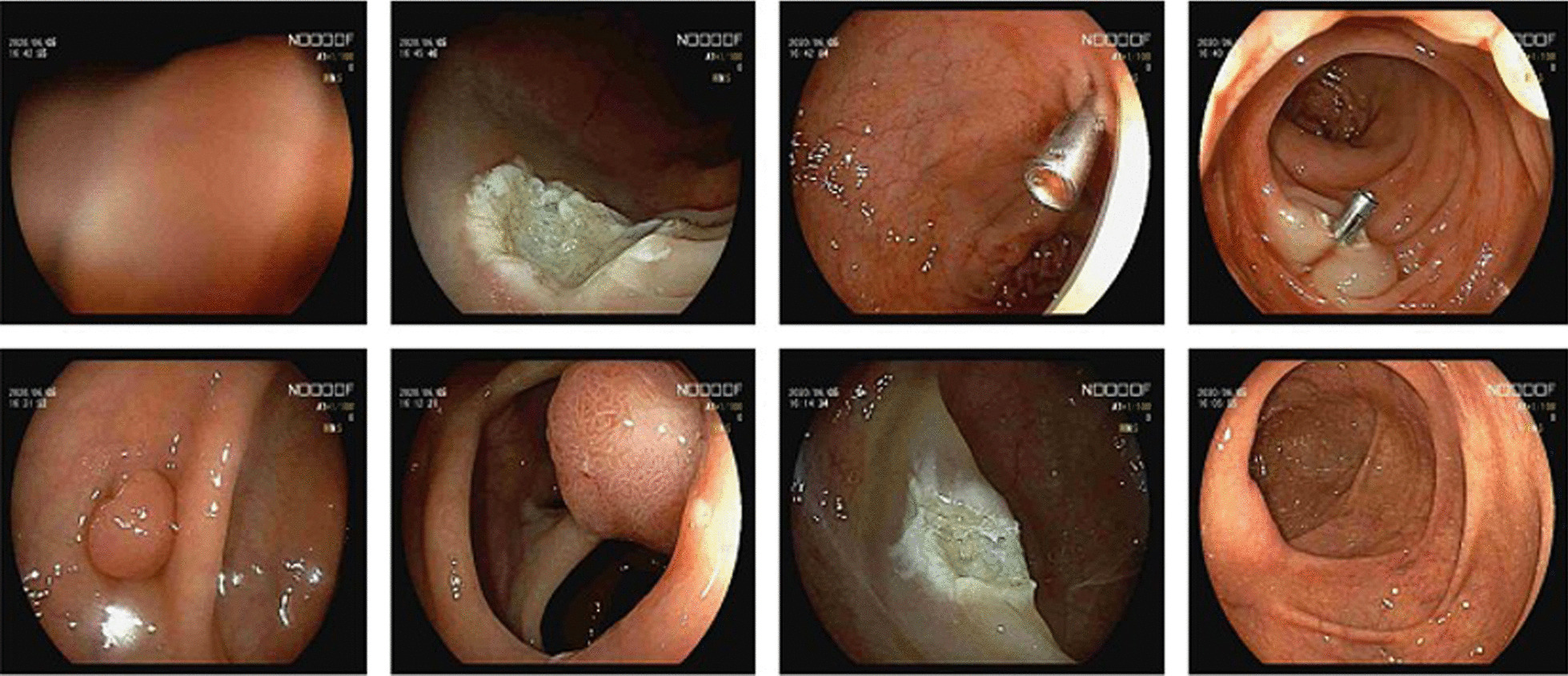
Suspected tumor lesions identified by colonoscope. Including four tumor lesions and chronic inflammations according to subsequent pathological evaluation

The patient is a 56-year-old Chinese man. He had diarrhea and hematochezia which lasted more than 10 days without obvious predisposing causes. The patient visited a hospital and received colonscopy of which the results were indicative of sigmoid colon cancer. Then, the patient was admitted to our hospital for further diagnosis and treatment. The patient self-reported no histories of hypertension, heart disease, diabetes and genetic diseases. There was no abnormality observed during physical examination. Tumors and bloodstain were not identified via digital rectal examination. The magnetic resonance imaging (MRI) and computed tomography (CT) of the abdomen indicated wall thickening of the sigmoid colon. Polyps were observed at the hepatic flexure of ascending colon and transversum colon (40 cm and 35 cm from anal verge respectively) via colonoscopy. In addition, the colonoscopy also identified suspected tumors at sigmoid colon and rectum (18 cm, 14 cm and 7 cm from anal verge respectively). Tissue samples from above sites were retrieved through colonoscopy (Fig. 1) for pathological diagnosis.

**Fig. 2:**
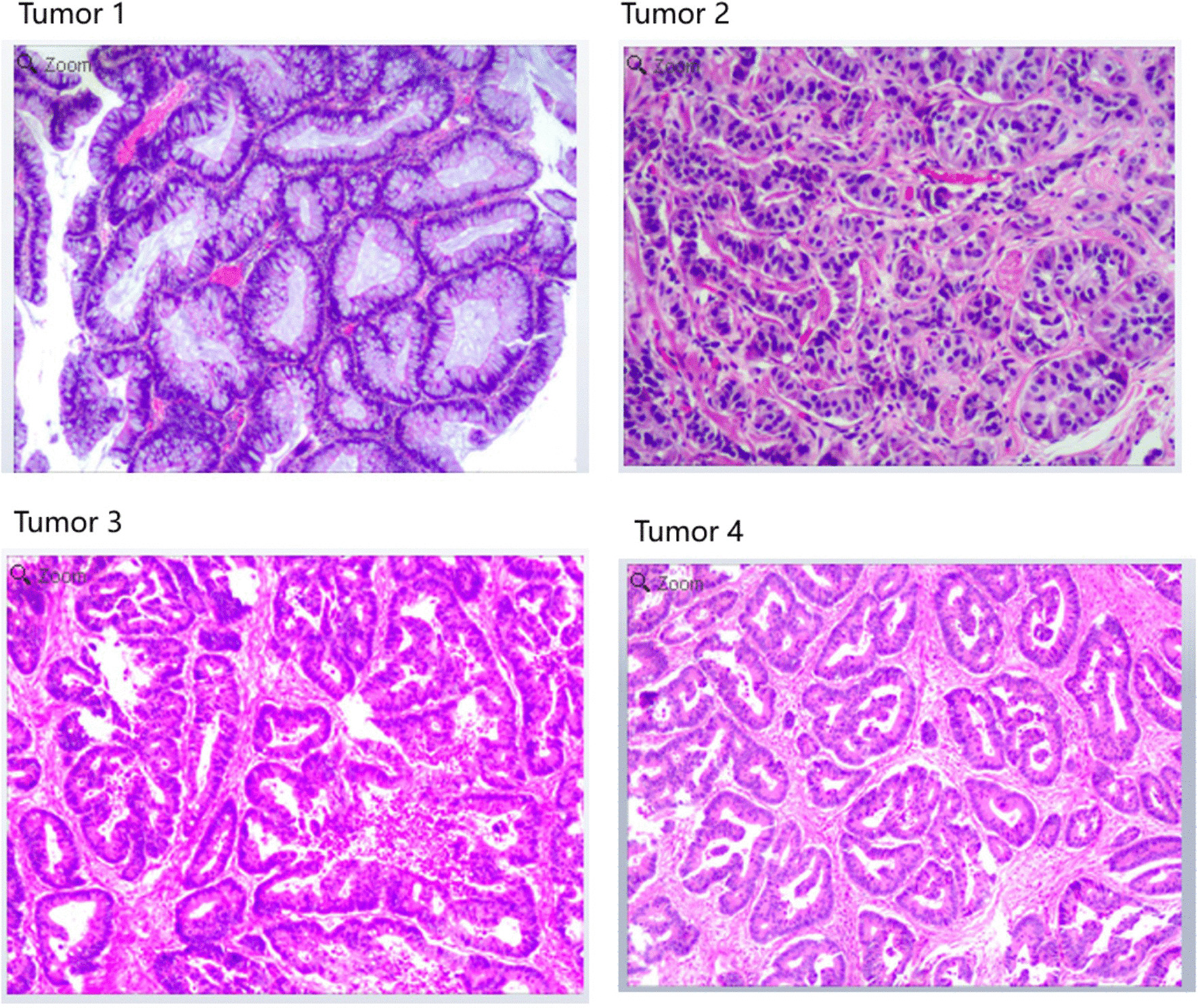
Tumor 1 is high-grade intraepithelial neoplasia at ascending colon. Tumor 2 is G1 NET at rectum. Tumor 3 is adenocarcinomas with moderate differentiation at sigmoid colon. Tumor 4 is adenocarcinomas with moderate differentiation at rectum

The tumor 1 located at the hepatic flexure of ascending colon was a high-grade intraepithelial neoplasia. The tumor 2 was located at rectum (7 cm from anal verge) with a size of 0.4*0.3 cm. The tumor tissue could be observed in mucosa and submucosa, but definite mitosis was not observed. According to immunochemistry results (CD56(+), CgA(+), Syn(+), Ki-67(< 2%+), CDX2(weak+), CK(+), CK20(−), SSTR-2(+)), the tumor 2 was a neuroendocrine tumor (NET) of grade 1 (G1). The tumor 3, located at sigmoid colon (18 cm from anal verge) with maximum diameter of approximately 4 cm, was an adenocarcinoma with moderate differentiation. Muscular layer infiltration was observed with a minimum distance to serosa less than 1 mm, while blood vessel invasion and nerve infiltration were not observed. The tumor 4, located near the rectum (14 cm from anal verge) with maximum diameter of approximately 1 cm, was also an adenocarcinoma with moderate differentiation. Muscular layer infiltration and suspicious blood vessel invasion were observed, but nerve infiltration was not observed. The other two samples from transversum colon indicated chronic inflammation with polypoid and local lymphoid hyperplasia (40 cm from anal verge) and tubular adenoma with low-grade intraepithelial neoplasia (35 cm from anal verge) respectively (Fig. 2).

**Fig. 3:**
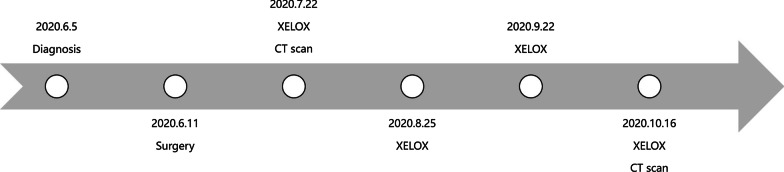
Treatment summary of the patient from diagnosis to last follow-up

The patient received laparoscopic radical resection of sigmoid carcinoma and partial rectum resection. Tissue samples of these four tumors were sent for capture-based targeted sequencing (Table 1). The tumor 1 (70% tumor content) had following mutations: *KRAS* p.G13D (allelic fraction (AF) = 35.27%), *APC* p.R283*(AF = 32.94%) and *PIK3CA* p.E545K(AF = 4.34%). The tumor 2 (NET G1, 10% tumor content) had mutations in *APC* p.S1315*(AF = 25.81%). The tumor 3 (pT3N0M0, stage IIA, 60% tumor content) harbored *ERBB2* amplification (CN = 3.0), *APC* p.S1400*(AF = 40.52%), *APC* p.R302*(AF = 17.90%), *PIK3CA* p.E542K(AF = 21.72%) and *TP53* p.A159V(AF = 58.79%). The tumor 4 (pT1N0M0, stage I, 3% tumor content) harbored mutaitons in *APC* p.R232*(AF = 2.32%) and *TP53* p.T125*(AF = 2.90%). Microsatellite status was evaluated and all tumors had microsatellite stable (MSS). After the surgery, the patient was treated with XELOX based adjuvant chemotherapy (oxaliplatin 200 mg d1 ivggt, capecitabine 1.5 g bid d1-d14 po) for four cycles (Fig. 3). The process of chemotherapy was smooth without severe adverse effects. No abnormalities were observed in blood routine reexamination of the patient. Four months after surgery, CT scan of the patient indicated no recurrence.

**Table 1:** Genomic profiling results of each tumor sites

Tumor site		Pathology type	Tumor content (%)	Identified mutations and MSI status*
Tumor 1	Ascending colon	Intraepithelial neoplasia (high-grade)	70	KRAS p.G13D(35.27%) APC p.R283*(32.94%) PIK3CA p.E545K(4.34%) MSS
Tumor 2	Rectum	Neuroendocrine tumor (G1)	10	APC p.S1315*(25.81%) MSS
Tumor 3	Sigmoid colon	Adenocarcinoma (moderate differentiation)	60	ERBB2 amp (CN: 3.0) APC p.S1400*(40.52%) APC p.R302*(17.90%) PIK3CA p.E542K(21.72%) TP53 p.A159V(58.79%) MSS
Tumor 4	Rectum	Adenocarcinoma (moderate differentiation)	3	APC p.R232*(2.32%) TP53 p.T125(2.90%) MSS

*Allelic fractions are indicated in percentage

*MSS* microsatellite stable

## Discussion and conclusions

Our study reported a very rare SCRC case with quadruple primary tumor lesions and performed comprehensive evaluation on each tumor lesions at pathological and molecular levels. The pathological evaluation of four tumor lesions identified a high-grade intraepithelial neoplasia at ascending colon, a G1 NET at rectum and two adenocarcinomas with moderate differentiation at sigmoid colon and rectum respectively. NET is a less common tumor type that usually located in gastrointestinal tract, in particular the rectum. The metastasis of a G2 NET from rectum to liver was reported in another SCRC case [6]. Meanwhile, adenocarcinomas are more commonly seen in SCRC patients. As reported in other cases with triple [7, 8] or quadruple [9] SCRC, the adenocarcinomas were frequently identified in the sigmoid colon, ascending and descending colon respectively. The presence of various histology types in the same patient indicated that carcinogenesis underlying multiple tumor lesions of SCRC might be different.

In addition to pathological evaluation, our study observed hetrogenous molecular features of four tumor lesions in SCRC through NGS-based genomic profiling. For example, *APC* mutations were identified in all tumor lesions, but their locations in *APC* were different including R283 in tumor 1, S1315 in tumor 2, R232 in tumor 4 and S1400/R302 double mutations in tumor 3. *TP53* mutations were presented in tumor 3 and 4 with different variants. Mutations of *PIK3CA* were presented in tumor 1 and 3 with same variant p.E545K. Mutation of *KRAS* was only presented in tumor 1. In a previous study of paired tumor lesions from 10 European patients with SCRC, genetic heterogeneities characterized by dissimilar mutations and independent genetic origins were reported [3]. Another study in 20 Chinese SCRC patients observed very few mutations shared in paired tumor lesions [4]. Commonly seen mutations in CRC such as *KRAS, APC, PIK3CA* and *P53* have been identified in previously published studies [3, 4] as well as in our case. Other important molecular biomarker of CRC such as MSI has also been evaluated in our study. MSS were consistently observed in four tumor lesions of SCRC. In western populations, it was reported that MSI-high occurs more frequently in SCRC compared with solitary CRC [10]. However, the frequency of MSI-high in Japan SCRC patients are lower which indicated existing populational differences of MSI [11]. Given genetic heterogeneities observed in different studies, it was believed that multiple tumor lesions of SCRC might be driven by different molecular mechanisms and was developed independently.

Surgical resection is the primary treatment option for SCRC. The patient in our study received laparoscopic radical resection of sigmoid carcinoma and partial rectum resection. The implementation of surgical procedures for different SCRC cases are varied which depends on the location of the tumors and personal status of the patient[2]. The prognosis of SCRC remains controversial. Some studies reported poor prognosis of SCRC compared with solitary CRC. A recent study in Dutch population reported higher rates of complicated postoperative course, failure to rescue, and mortality in SCRC patients [12]. When compared with metachronous CRC, patients with SCRC also showed worse prognosis featured by higher rates of recurrence and cause-specific death [13]. Meanwhile, numbers of studies indicated no differences in survival when SCRC and solitary CRC patients have same tumor stage and curative resection [1, 2]. In addition to surgical resection, the use of targeted therapies for SCRC remains undefined. Considering heterogenous molecular profiles among different tumor lesions in SCRC, the potential actionable therapeutic targets might be varied [4]. The choice of treatment regiments for SCRC should consider independent patients as well as independent tumor lesions. The patient in our case received XELOX chemotherapy after surgical treatment and remained disease-free. Long-term follow-up are warranted to evaluate prognosis of the case.

Our study reported a very rare case with quadruple synchronous colorectal cancer. The four tumor lesions were subjected to pathological evaluation and comprehensive genomic profilling. Each tumor showed unique pathological and genetic features which were indicative of heterogenous molecular mechanisms underlying SCRC.

## Data Availability

Data and materials relavent to the study are included in this report. Further inquiries can be directed to the corresponding author.

## Abbreviations

SCRC: synchronous colorectal cancer
CRC: colorectal cancer
MRI: magnetic resonance imaging
CT: computed tomography
NET: neuroendocrine tumor
AF: allelic fraction
MSS: microsatellite stable
XELOX: oxaliplatin and capecitabine
